# Exponential vs Gaussian Correlation Functions in the Characterization of Block Copolymer Grain Structure by Depolarized Light Scattering

**DOI:** 10.1021/acs.macromol.3c01835

**Published:** 2023-12-26

**Authors:** Xin Wang, Jacob L. Thelen, Xiuhong Li, Nitash P. Balsara, Bruce A. Garetz

**Affiliations:** † Department of Chemical and Biomolecular Engineering, NYU Tandon School of Engineering, Brooklyn, New York 11201, United States; ‡ Department of Chemical and Biomolecular Engineering, University of California, Berkeley, California 94720, United States; § Environmental Energy Technologies Division, Lawrence Berkeley National Laboratory, Berkeley, California 94720, United States; ∥ Materials Sciences Division, Lawrence Berkeley National Laboratory, Berkeley, California 94720, United States

## Abstract

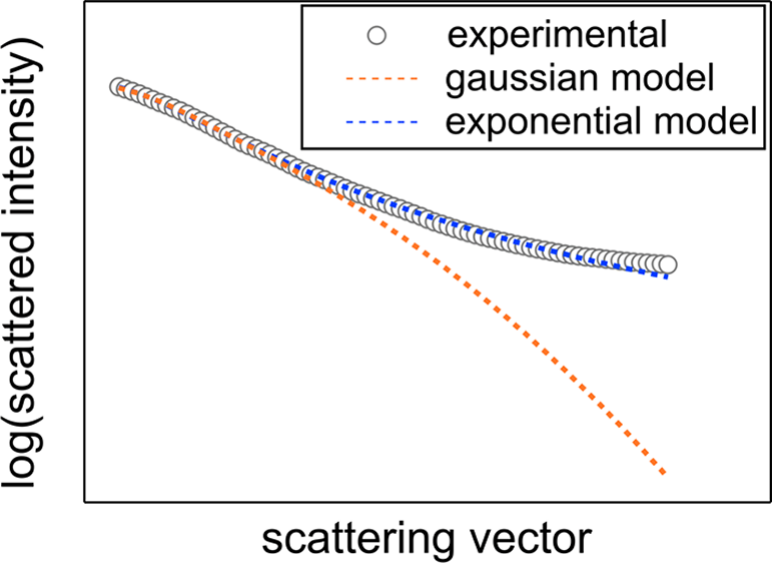

Block copolymer (BCP) grain structure affects the mechanical, optical, and electrical properties of BCP materials, making the accurate characterization of this grain structure an important goal. In this study, improved BCP grain parameters were obtained by employing an exponentially decaying correlation function within the ellipsoidal grain model, instead of the Gaussian correlation function that was used in previous work. The exponential correlation function provides a better fit to the experimental depolarized light scattering data, which outweighs the disadvantage that it requires numerical integration to obtain the model scattered intensity.

## Introduction

When cooled below the order–disorder transition (ODT) temperature in the absence of external fields, neat block copolymers (BCPs) and BCP/salt mixtures typically form randomly oriented micrometer-sized grains with concomitant defects.^1−8^ When the ordered phase consists of lamellae or cylinders, a grain exhibits form birefringence^9^ and behaves optically like a uniaxial crystal, with the optic axis perpendicular to the lamellae or parallel to the cylinder axes. Depolarized light scattering (DPLS) can be used to probe grain sizes of dimensions comparable to or greater than the wavelength of the probing light source.^9,10^ DPLS is not sensitive to the lamellar or cylinder size and spacing, which are several orders of magnitude smaller than the wavelength of visible light. DPLS has been demonstrated to be an effective and complementary tool to small-angle X-ray scattering,^11^ small-angle neutron scattering,^12^ polarized light microscopy (POM),^11,13,14^ and electron microscopy^15^ to study the thermodynamics and kinetics of grain growth. DPLS enabled one of the earliest studies of grain growth and defect annihilation in BCPs,^16^ paving the way for later research in this area.^5,17^ Such studies are important because the BCP grain structure affects the mechanical, optical, and electrical properties of BCP materials.^11,12,15,18−20^

Early efforts to model the DPLS patterns arising from polymer films were pioneered by Stein and co-workers in the 1960s and 1970s.^21^ They were able to estimate the average size of spherulites in semicrystalline polymer films of low-density polyethylene based on the angular spread of four-leaf clover-type depolarized scattering patterns that they recorded photographically.^22^ Their studies also included an early report of an X-shaped depolarized scattering pattern from a styrene–butadiene–styrene BCP film.^22,23^

The theoretical framework for relating a DPLS scattering pattern to the granular organization of the material is critical to the extraction of grain-structure parameters. The models we have used over the past 30 years were developed to explain the experimental observations encountered during that period.^10,24−28^ Initially, we were trying to understand the observation that quiescently ordered BCP samples held between crossed polarizers transmitted a small amount of incident light. This led to the development of the “slab” model published in 1992.^9^ This model assumes that the incident light passes through a sequence of grains, with randomly oriented optic axes, as it propagates through the sample, with each grain treated as a slab with transverse dimensions much greater than the wavelength of the light. The model thus ignores diffraction and treats the changes in polarization that occur as the light travels through a series of slabs as a random walk in the polarization state, allowing the estimation of the average longitudinal thickness of a grain. Shortly thereafter, we observed that these same lamellar samples, in addition to depolarizing the incident light, also diffracted the light, producing an azimuthally symmetric far-field scattering pattern. This led to the development of a spherical grain model (SGM) in which the probability that two points in the sample were located in the same grain was described, on average, by a spherically symmetric Gaussian correlation function. The diffracted intensity was proportional to the spatial Fourier transform of this correlation function, allowing an estimation of the average transverse dimension of a grain.^10,25^ In a later study of similar samples, we analyzed transmission electron microscopy (TEM) images to obtain this correlation function directly in position space, corroborating the grain sizes predicted by the analysis of DPLS patterns in reciprocal space. That study also revealed that the correlation function decayed exponentially with increasing distance between two points in the sample rather than with a Gaussian functional dependence, although both the exponential and Gaussian correlation functions produced indistinguishable fits to the DPLS intensity profiles.^24^

We later studied a series of cylindrical BCP samples that exhibited 4-fold-symmetric “X” or “cloverleaf” scattering patterns. By “cloverleaf”, we mean a 4-fold “X” pattern, with lobes at 45° to the polarizer axes, but with four intensity maxima that are at an angle away from the forward direction. The ellipsoidal grain model (EGM) was developed to explain such “X” patterns.^25,28^ Because any single grain model predicts a maximum intensity in the forward direction, the modeling of “cloverleaf” patterns required the development of the correlated ellipsoidal grain cluster model.^25,28^ Later POM studies by Lodge and co-workers that imaged the microscopic grain structure in BCP solutions reported the presence of ellipsoidal grains as well as spherulites and other types of clusters.^13,14,29,30^ In retrospect, our grain cluster models consisting of three mutually orthogonal ellipsoidal grains to model “cloverleaf” patterns can be considered “minimalist” models for a 3D BCP spherulite^25^

For quiescently quenched samples (not subjected to external forces), the recorded DPLS patterns always exhibit either azimuthal or 4-fold angular symmetry. This is true both in the early stages of grain growth, when grains are surrounded by disordered regions of the sample, and at later times, when grains impinge on each other and are separated by defect regions, so that the same models and fitting procedures can be used in both cases.^16^

We have also recorded and analyzed DPLS patterns from a BCP sample subjected to reciprocating shear flow.^31^ In this case, the grain orientation distribution is no longer isotropic, and the scattering patterns no longer exhibit azimuthal or 4-fold symmetry but have a lower 2-fold angular symmetry. Such samples can be characterized as being composed of single-crystal and granular volume fractions. The current paper concerns only quiescently quenched samples.

The EGM has withstood the test of time and has been used in nearly all subsequent studies of DPLS in BCPs. We have used it to study BCP/Li^+^ mixtures that have application as lithium battery electrolytes.^18−20,28,32−35^ The patterns obtained from BCP/salt mixtures are qualitatively identical to patterns obtained from neat BCP samples, as the lithium salt is dissolved in the BCP. While the dissolved salt will change the refractive indices of the grains and therefore the overall intensity of the scattering pattern, it does not change its angular spread, which determines the grain parameters obtained. In 2014, we published analytic expressions for the intensity distribution associated with the single EGM using a Gaussian correlation function, which led to a faster, more convenient least-squares fitting of grain parameters from experimental scattering data.^28^ Most recently, the single EGM has been used in studies involving the propagation of circularly and elliptically polarized light through a BCP sample.^26,27^

The use of and justification for an exponential correlation function dates back to the work of Debye and co-workers in the late 1940s and 1950s, who employed it to fit the light scattering curves from inhomogeneous solids such as Lucite and glass.^36^ They showed that an exponential correlation function could be derived theoretically for porous materials by assuming a distribution of pores with random sizes and shapes.^37^

As mentioned above, although our TEM analysis revealed that an exponential correlation function was more realistic that a Gaussian one,^24^ we have continued to use the Gaussian correlation function mainly because of its convenience. The calculation of scattered intensities from the exponential correlation function requires numerical integration over the azimuthal and polar angles that describe the orientation of the grain optic axis. The purpose of this paper is to establish the procedure for incorporating an exponential correlation function into the analysis of the DPLS profiles obtained from quiescently quenched BCPs or BCP/salt mixtures.

In this paper, we calculate the theoretical scattered intensity as a function of scattering angles and grain parameters using numerical integration, based on the single EGM with an exponentially decaying correlation function. We use these values to least-squares fit several experimentally obtained DPLS patterns from BCP/Li^+^ mixtures to obtain values of grain-size parameters  and *w*. We compare these fits to those obtained using a Gaussian correlation function. We show that the exponential fits are generally superior to the Gaussian fits, especially for large values of *q* and for samples that exhibit nearly azimuthally symmetric “O” scattering patterns.

## Theory

**Figure 1:**
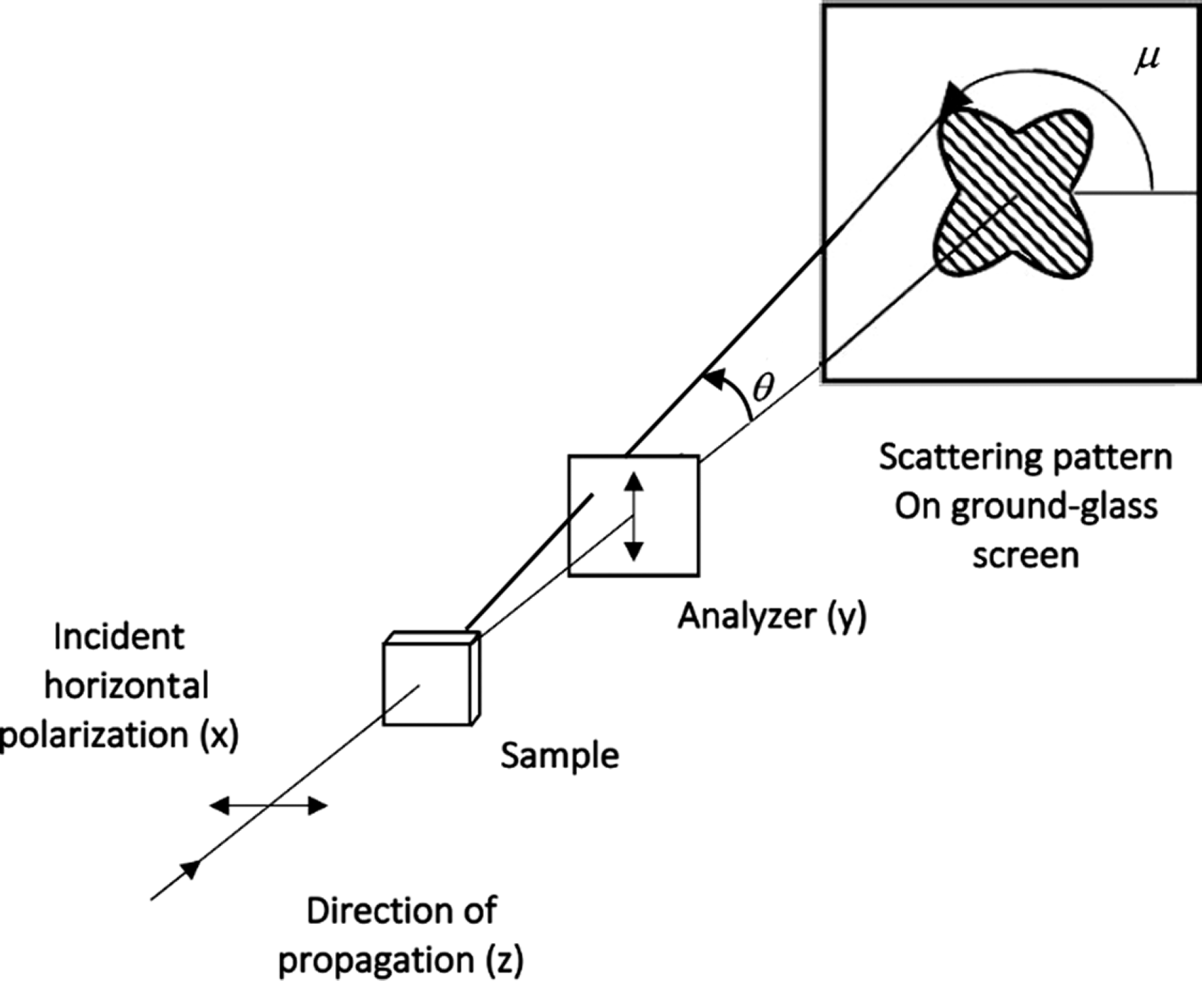
Schematic of the optical setup showing various directions and angles.

Figure 1 shows a BCP/Li^+^ sample consisting of randomly oriented uniaxial grains being illuminated by a collimated x-polarized laser beam propagating in the *z*-direction.^9^ An analyzer transmits the *y*-polarized component of the scattered light, which impinges on a ground glass plate, and a digital camera records the intensity at each pixel as a dimensionless number between 0 and 255. We denote this dimensionless intensity as *I*(*q⃗*), where *q⃗* is the scattering vector, whose magnitude is given by , where θ is the polar scattering angle shown in Figure 1, and λ is the wavelength of the incident light.

**Figure 2:**
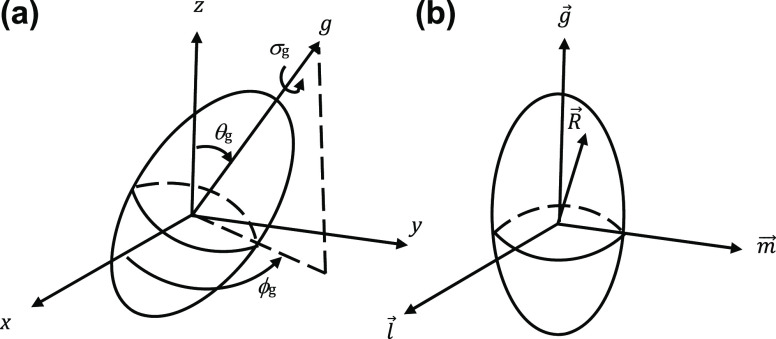
(a) Grain orientation angles in terms of laboratory coordinate system; (b) grain-centered coordinate system.

We have shown in ref (27), eq 6 and ref (25), eq 13 that, for a collection of uncorrelated ellipsoidal grains with the shape axis coincident with the optic axis, *I*(*q⃗*) can be expressed as
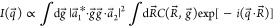
1where *a⃗*_1_ and *a⃗*_2_ are the unit vectors describing the polarization states of the incident and scattered rays, respectively, *R⃗* ≡ *r⃗* – *r⃗*′, where *r⃗* and *r⃗*′ are vectors representing two different positions in the sample, *g⃗* is a unit vector in the direction of the optic axis of the grain, and *C*(*R⃗*, *g⃗*) is a correlation function that represents the probability that the two points separated by *R⃗* are both in the same grain. The notation  represents an integration over all the angles that define the orientation of a grain, where θ_g_, ϕ_g_, and σ_g_ are the polar, azimuthal, and spin angles of *g⃗*, respectively, as shown in Figure 2a. If the grain is, on average, an ellipsoid of revolution, then *C*(*R⃗*, *g⃗*) is independent of the spin angle, σ_g_. In addition, if the incident light is *x*-polarized and the analyzer selects *y*-polarized scattered light, then *a⃗*_1_ = *x̂* and *a⃗*_2_ = *ŷ*, and the transmission factor |*a⃗*_1_^*^ · *g⃗g⃗* · *a⃗*_2_|^2^ is equal to , yielding
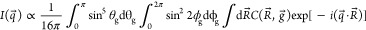
2

The *q⃗*-dependence of the scattered intensity of a single grain is thus given by the spatial Fourier transform of the correlation function *C*(*R⃗*, *g⃗*), and the scattered intensity of a collection of randomly oriented uncorrelated grains is obtained by integrating over all possible orientations of the optic axis, *g⃗*, weighted by the transmission factor for each grain orientation through the crossed polarizers.

The current version of the EGM assumes that the correlation function falls off monotonically as the distance between the two points in the sample increases according to a Gaussian functional dependence:
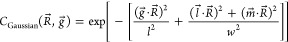
3

{*g⃗*, *l⃗*, *m⃗*} form an orthogonal set of unit vectors (see Figure 2b), and  and *w* are grain size parameters along and perpendicular to the optic axis, respectively. This correlation function has the property that surfaces of constant *C*(*R⃗*, *g⃗*) are ellipsoids of revolution. In particular, the surface with *C*(*R⃗*, *g⃗*) = 1/*e* is an ellipsoid of revolution with semimajor axis  and semiminor axis *w*.

This choice of *C*(*R⃗*, *g⃗*) was made because it resulted in analytic expressions for the scattered intensity.^10^ This allows the efficient least-squares fitting of DPLS data to the model to extract average grain lengths and widths. On the other hand, our early experimental study of orientation correlations in BCPs from the analysis of TEM images suggested that the correlation function falls off exponentially with increasing *R*.^24^ When an exponential correlation function is employed with the single EGM, the resulting integrals cannot be evaluated analytically and a two-dimensional numerical integration over polar and azimuthal angles of grain orientation is required.

The only exponentially decaying correlation function whose surfaces of constant *C*(*R⃗*, *g⃗*) are ellipsoids of revolution is
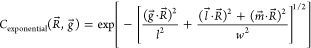
4

The surface with *C*(*R⃗*, *g⃗*) = 1/*e* is an ellipsoid with semimajor axis  and semiminor axis *w*, just as with the Gaussian correlation function shown in eq 3.

Evaluation of the spatial Fourier transform of these correlation functions, which is given by the integral ∫d*R⃗ C*(*R⃗*, *g⃗*) exp[−*i*(*q⃗* · *R⃗*)], yields the expressions shown in eqs 5 and 6:
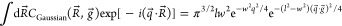
5
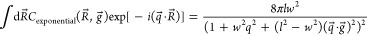
6

It is worth discussing the connections between the pairs of grain parameters,  and *w*, used in eqs 3 and 4, respectively. The concept of a grain in BCPs is modeled on the grain structure of crystalline solids, such as metals, which consist of many small single crystals with a range of sizes and random orientations separated by grain boundaries. In BCPs, a grain is a small, ordered region in which the microstructure (e.g., lamellae or cylinders) is coherent. BCP grains are sometimes separated by defect boundaries, but in some cases, the lamellae or cylinders can curve continuously through large angles so that the concept of a BCP grain is partly fictitious. Nevertheless, an average grain size is still a useful construct in the BCP samples. For convenience, we define the average grain boundary to be the ellipsoidal surface on which the grain correlation function, *C*(*r*), falls to 1/*e* of its maximum value of unity at the grain center. Since the Gaussian correlation function decays considerably faster than the exponential one, one should not expect the best-fit values of  and *w* using each correlation function to be equal to each other. In the special case that  = *w*, if we equate the first moments, ⟨*R*⟩, calculated using the two correlation functions, we find that *w*_E_ = 2*w*_G_/(3π^1/2^) ≈ 0.6*w*_G_, where *w*_E_ and *w*_G_ are the grain widths obtained using the exponential and Gaussian correlations functions, respectively.

In the paraxial limit (small θ),
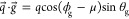
7

so that the integral that must be evaluated numerically is
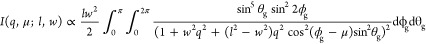
8

In ref (28), we showed, based on symmetry arguments, that the DPLS scattered intensity from a collection of randomly oriented ellipsoidal grains with the optic axis parallel to the shape axis can be written as the sum of an azimuthally symmetric term and a term with 4-fold symmetry in μ:
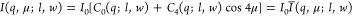
9where *I*_0_ is the intensity in forward direction, *C*_0_(*q*; *l*, *w*) dictates the overall decay of scattered intensity as a function of *q,* and *C*_4_(*q*; *l*, *w*) is a measure of the depth of the 4-fold angular modulation of the scattered intensity. *C*_0_(*q*; *l*, *w*) is normalized so that it is equal to unity when *q* = 0. *C*_0_(*q*; *l*, *w*) and *C*_4_(*q*; *l*, *w*) have different functional forms for different correlation functions. *I̅*(*q*, μ; *l*, *w*) is a normalized scattered intensity such that it is equal to unity when *q* is zero. For a Gaussian correlation function, the *C*_0_ and the *C*_4_ components have analytical expressions shown in eqs 10 and 11(28)
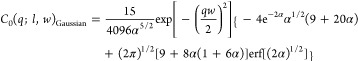
10
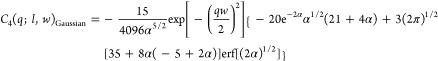
11where . In the limit of α → 0, *C*_0_ and *C*_4_ can be expressed in terms of power series in α:
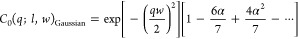
12
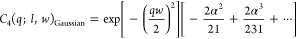
13

In contrast, the *C*_0_ and *C*_4_ components for the exponential correlation function must be evaluated numerically using eq 8. However, in the limit that  is much smaller than unity, the scattered intensity can be expanded in a power series in β:
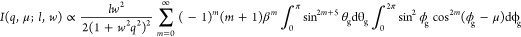
14

and *C*_0_(*q*; *l*, *w*) and *C*_4_(*q*; *l*, *w*) can be expressed as
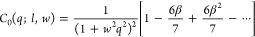
15
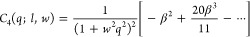
16

In the limit of either α or β = 0, we have the special case that *l* = *w*, so that the EGM reduces to a SGM, which is characterized by a single size parameter, *w*. For the exponential correlation function, we obtain the analytic expressions  and *C*_4_(*q*; *l*, *w*) = 0. For the Gaussian correlation function, we obtain the expressions  and *C*_4_(*q*; *l*, *w*) = 0.

*I*_0_, , and *w* are parameters that can be least-squares fit to the experimental DPLS scattering pattern. An azimuthally symmetric “O” scattering pattern is a common type of pattern that has been observed in many previous studies, and in theory, a perfectly azimuthally symmetric pattern indicates that  = *w* and results in the *C*_4_ component equal to zero for all *q* values. A 4-fold symmetric “X” pattern as shown in Figure 1 is another common type of scattering pattern, which indicates  / *w* ≫ 1, and results in a *C*_4_ component that is negative for all *q* values.^28^ The details of the extraction of the grain parameters from the experimental scattering patterns are covered in the DPLS data reduction and analysis section.

## Experimental Section

### Materials and Sample Preparation

The polystyrene-*b*-poly(ethylene oxide) (SEO) diblock copolymer used in this study was synthesized, purified, characterized, and finally doped with bis(trifluoromethanesulfonyl)imide (LiTFSI) as described in ref (28). By blending the SEO with LiTFSI, we obtained a BCP mixture, SEO (1.7–1.4) with a salt concentration of *r* = 0.075, where *r* is the ratio of Li^+^ ions to ethylene oxide monomer units, and 1.7 and 1.4 are the number-averaged molecular weights of the polystyrene (PS), and poly(ethylene oxide) (PEO), blocks in kg mol^–1^, respectively. When it was cooled below the ODT temperature, this sample formed a lamellar nanostructure. In this study, we use “SEO” as an abbreviation for the BCP mixture of SEO(1.7–1.4), *r* = 0.075. The electrolyte mixture was loaded into a custom-built aluminum sample holder with fused silica windows as described in ref (28) and was shipped from Berkeley to Brooklyn for the light scattering studies.

### DPLS and Birefringence Measurements

The digital images of scattering patterns analyzed in this study were acquired during the same experimental runs conducted to generate the scattering patterns reported in ref (28). In one of the experiments, the SEO sample with an order–disorder transition temperature of 124 ± 2 °C was heated to 140 °C (16 °C above *T*_ODT_), then quenched to 112 °C. The resulting image is referred to in terms of the corresponding quench depth of 12 °C below the ODT temperature. In another experiment, the quench depth was 20 °C below the ODT temperature. The charge-coupled device camera was set to capture one scattering pattern every minute in the first hour, starting at the moment when the quench began. The scattering patterns were stored in the form of 480 × 640 pixel TIFF image files. The intensity at each pixel was recorded as a dimensionless number between 0 and 255.

## Computational Methods

*I̅*(*q*, μ; *l*, *w*) was computed for the exponential correlation function by the numerical integration of eq 8. *C*_0_(*q*; *l*, *w*) and *C*_4_(*q*; *l*, *w*) values were calculated by the numerical integration of eqs 17 and 18.
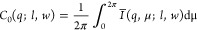
17
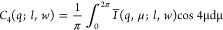
18

These two functions were used in the least-squares fitting of the experimental scattering patterns to the exponential EGM. For given values of *q*, *l*, and *w*, *I̅*(*q*, μ; *l*, *w*) was calculated by numerical integration of eq 8 for an array of 201 μ values ranging from 0 to 2π in steps of π/100. Then *C*_0_(*q*; *l*, *w*) and *C*_4_(*q*; *l*, *w*) values were calculated by numerical integration of eqs 17 and 18.

## Data Reduction and Analysis

**Figure 3:**
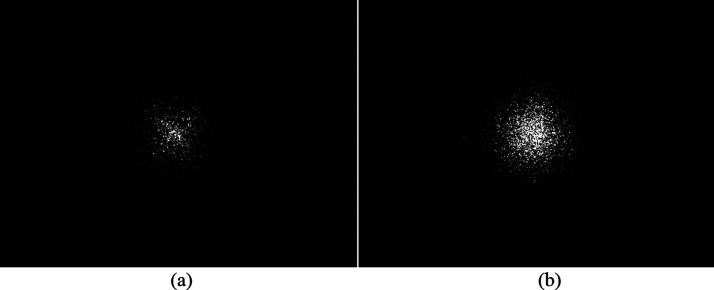
Parts (a) and (b) represent “X” and “O” scattering patterns obtained at quench depths of 12 and 20 °C, respectively. The maximum scattering vector *q* at the sides of each image is 1.13 μm^–1^. The contrast of the two patterns was identically adjusted to enhance major features that were not clear in the original lower-contrast patterns.

As seen in Figure 3, two scattering patterns were selected to represent an “X” pattern and an “O” pattern, which are associated with two populations of grains with very different shape anisotropies^26,28^ The shape anisotropy of a given sample depends on its composition and its thermal history, such as the quench depth and the time since the initiation of the quench. The “X” pattern in Figure 3a shows grains with large aspect ratios,  /*w*, while the “O” pattern shows grains with aspect ratios closer to unity.^8^ The speckle seen in the two light scattering patterns arises from the interference of radiation scattered from different pairs of grains in the sample. While speckle is not noise *per se*, it is noise with respect to the EGM, which models only the incoherent component of the scattered intensity. Following the same preprocessing procedure used in ref (28), the original TIFF images were convoluted with a Gaussian low pass filter with an fwhm of 12 pixels to eliminate the high spatial-frequency components from the speckle patterns before the extraction of the experimental *f*_0_ and *f*_4_ cosine moments using eqs 19 and 20:
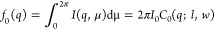
19
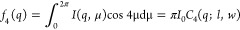
20where *I*(*q*, μ) is the processed experimental DPLS scattering pattern. The 12-pixel filter was chosen to remove as much speckle as possible without losing information about the incoherent portion of the scattered intensity. The extracted *f*_0_ and *f*_4_ cosine moments were then least-squares fit to both the Gaussian and exponential *C*_0_(*q*; *l*, *w*) and *C*_4_(*q*; *l*, *w*) functions, respectively.

**Figure 4:**
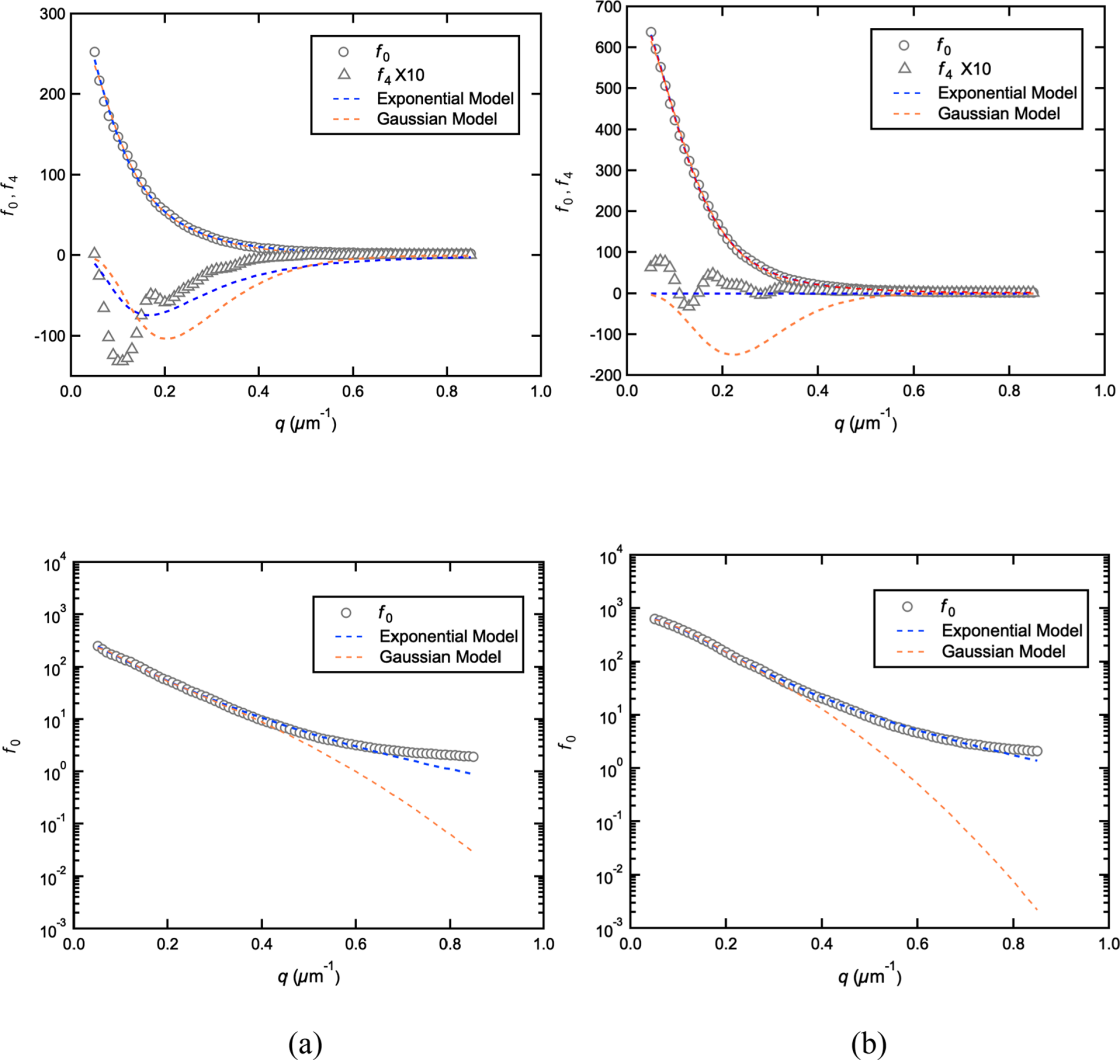
Least-squares fits (dashed lines) of cosine moments (symbols) experimentally extracted from the scattering patterns in Figure 3. Parts (a) and (b) represent the fits of “X” and “O” patterns, respectively. Lower plots are semilog plots of *f*_0_ vs *q*.

Figure 4 shows the experimental *f*_0_ and *f*_4_ cosine moments (represented by symbols “O” and “Δ”, respectively) extracted through the numerical integration of eqs 19 and 20, evaluated at 81 values of *q* from 0.05 to 0.85 μm^–1^, for both the “X” and “O” patterns. The oscillations in both of the *f*_4_ cosine moments arise from low-frequency speckle features in the scattering patterns that have not been filtered out by the low-pass filter. The Levenberg–Marquardt method was used to perform a nonlinear least-squares fitting to both the Gaussian and exponential *C*_0_(*q*; *l*, *w*) and *C*_4_(*q*; *l*, *w*) functions. The blue and orange dashed lines in Figure 4 represent the curves computed with the corresponding best-fit grain parameters given in Table 1, associated with the exponential and Gaussian correlation functions, respectively. All data points of the experimental *f*_0_ and *f*_4_ functions were weighted equally to obtain the best-fit parameters shown in Table 1.

**Table 1:** Best-Fit Grain Parameters for the Least-Squares Fitting of the Two Models

pattern	parameter	Gaussian EGM	exponential EGM
X	*I*_0_	45 ± 1.2	50 ± 1.2
	*w* (μm)	5.9 ± 0.3	3.1 ± 0.1
	*l*/*w*	4.3 ± 0.3	3.6 ± 0.2
	χ^2^ (min)	11.3	5.4
O	*I*_0_	111 ± 2	116 ± 0.8 (116 ± 0.6)^a^
	*w* (μm)	7.5 ± 0.3	5.5 ± 7.9 (5.5 ± 0.02)^a^
	*l*/*w*	2.7 ± 0.2	1.0 ± 3.4^a^
	χ^2^ (min)	48.4	5.3 (5.7)^a^

a The uncertainties associated with the grain parameters *w* and *l*/*w* get large as *l*/*w* approaches unity. See text for explanation. The parameters and uncertainties in parentheses were obtained by fitting the same experimental data to the SGM for which *l*/*w* ≡ 1.

For both scattering patterns, both the Gaussian and exponential EGMs appear to do a good job of fitting the *f*_0_ curve, although when one takes a closer look using a semilog plot, the Gaussian model consistently underestimates *f*_0_ in the tail of the curve, for *q* values between 0.4 and 0.85 μm^–1^. For the “X” pattern, both the Gaussian and exponential EGMs predict the correct shape and negative sign of the *f*_4_ curve, but the position and depth of the minimum are not well estimated. It is not uncommon to see this combination of a good-fit on *f*_0_ and a poor-fit on *f*_4_ in previous studies on BCP or BCP/salts mixtures.^11,26−28^ The Gaussian model does a better job of getting the depth of the minimum, while the exponential model does a better job of getting the *q*-location of the minimum. The mismatches of *f*_4_ seen in Figure 4 are enhanced because the *f*_4_ curve is magnified by a factor of 10 relative to *f*_0_. As we have pointed out in the past, given that the single EGM has only two parameters (*w*, *l*/*w*) that affect the shape of the curves (*I*_0_ is just a scaling factor), it does a remarkable job of exhibiting the essential features of the DPLS pattern, despite the fact that actual BCP samples have a very complex grain structure, as seen in TEM imaging. The superiority of the exponential EGM is also apparent in the fits to the “O” pattern. The Gaussian EGM predicts a fairly deep minimum in *f*_4_ that is not reflected in the experimental data, which exhibit small oscillations around zero. On the other hand, the exponential model estimates *f*_4_ to be close to zero for all values of *q*, in agreement with the experimental data. As seen in Table 1, for both the “X” and “O” patterns, the value of χ^2^(min) is smaller for the exponential fit than for the Gaussian fit, also indicating the superiority of the exponential EGM.

When we compare the magnitudes of *w* obtained from the Gaussian and exponential least-squares fits, we see that the exponential model gives *w* values that are a factor of 0.5–0.7 the size of *w* obtained from the Gaussian model. This is consistent with the factor of 0.6 based on theoretical considerations derived in the Theory section of this paper, as well as the results in ref (24). The computation time taken to perform the least-squares fitting to obtain grain parameters with the exponential model is about a hundred times longer than that required with the Gaussian model.

**Figure 5:**
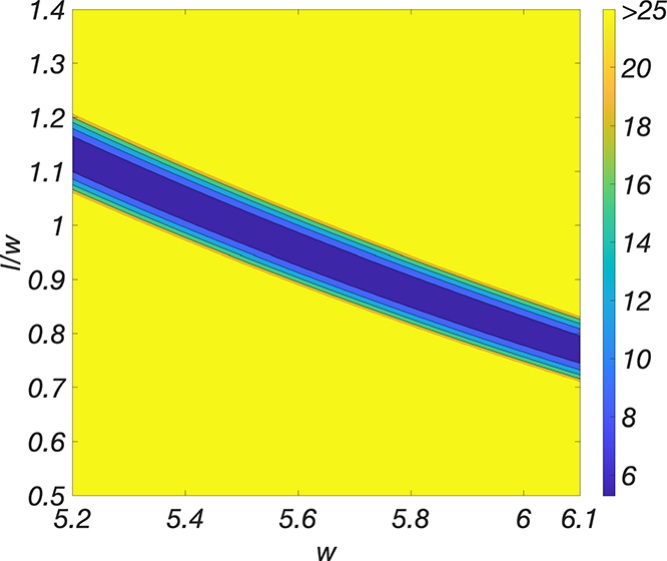
Contour plot of χ^2^ in the vicinity of the minimum for the exponential EGM fit of the “O” pattern, with *I*_0_ fixed at its best-fit value of 116. The color indicates the value of χ^2^ for a given value of *w* and *l*/*w*.

In the Levenberg–Marquardt method, uncertainties in the fitted model parameters are equal to the diagonal elements of the covariance matrix of the standard errors of the parameters. The covariance matrix is the inverse of the Hessian (second derivative) matrix for χ^2^ with respect to the three model parameters. Using the first terms in the power-series expansions for *C*_0_ and *C*_4_ in terms of α and β for the Gaussian and exponential models, respectively, one can show that the Hessian matrix is singular when *l*/*w* = 1 for both models. Therefore, the Hessian matrix cannot be inverted, and the covariance matrix does not exist; therefore, one cannot compute uncertainties for the model parameters. In addition, as *l*/*w* approaches unity, the calculated uncertainties approach infinity and are meaningless. This issue is related to the shape of the least-squares minimum as a function of the grain parameters. Rather than having a paraboloidal shape, the minimum is shaped like a narrow trough, as shown by the contour plot shown in Figure 5. In spite of this limitation, it is clear from the fits shown in Figure 4 and the minimum χ^2^ values shown in Table 1 that the exponential model is better at fitting the “O” pattern than the Gaussian model. A singular Hessian matrix is often associated with a model with more parameters than are needed to fit the data.^38^ An alternative for handling scattering patterns for which *l*/*w* is very close to 1 is to fit the data to the 2-parameter (*I*_0_, *w*) SGM described in the text below eq 16. When we fit the “O” pattern to this 2-parameter model, we get the parameters shown in parentheses in Table 1, which are nearly identical to the 3-parameter values, but the uncertainties are much smaller. If we were to plot the resulting theoretical curves in Figure 4b, they would be indistinguishable from the blue 3-parameter curves.

## Conclusions

In this paper, we have modified the EGM to incorporate an exponentially decaying correlation function instead of a Gaussian one for the characterization of ordered BCPs and BCP/salt mixtures. We have compared the least-squares fits of several experimentally obtained DPLS patterns from a BCP/salt mixture using these two types of correlation functions and find that the exponential correlation function does a better job of fitting the experimental data. While both functions give reasonable fits for the zeroth cosine moment *f*_0_ for small values of *q*, the exponential function does a much better job of fitting *f*_0_ for large *q* values. While both functions do a reasonable job of fitting the fourth cosine moment, *f*_4_, for an “X” scattering pattern, the Gaussian function does a poor job of fitting *f*_4_ for an “O” pattern. In both patterns studied, the exponential function exhibited a smaller χ^2^ value at the minimum of the least-squares fit. The only downside of the EGM with both the exponential and Gaussian correlation functions is that the uncertainties in the fitted values of *w* and *l*/*w* cannot be calculated for “O” patterns when *l*/*w* is close to unity, owing to the singularity of the Hessian matrix. We have shown that this can be resolved by using a 2-parameter SGM in such cases. We conclude that the use of an exponential correlation function is a significant improvement to the EGM and outweighs the added complication and increased computation time associated with performing the numerical integration required to evaluate the theoretical *C*_0_(*q*; *l*, *w*) and *C*_4_(*q*; *l*, *w*) functions.^31,36,37^
